# A novel activating role of SRC and STAT3 on HGF transcription in human breast cancer cells

**DOI:** 10.1186/1476-4598-6-69

**Published:** 2007-10-29

**Authors:** Michelle R Sam, Bruce E Elliott, Christopher R Mueller

**Affiliations:** 1 Department of Pathology and Molecular Medicine, Queen's University, Kingston, Canada; 2 Department of Biochemistry, Queen's University, Kingston, Canada; 3 Queen's Cancer Research Institute, Queen's University, Kingston, Canada

## Abstract

We have previously determined that the HGF promoter can be transactivated by a combination of activated Src and wild-type Stat3 in the mouse breast cell lines HC11 and SP1. To determine if this pathway is of relevance for the human disease, a series of human breast and other human cells lines were examined, and the status of key proteins in these cells determined. All of the human breast cell lines exhibited strong transactivation by a combination of activated Src and Stat3. This activation was dependent on a Stat3 recognition element present at nt-95. The exception was the ErbB2 over-expressing cell line SK-BR-3 where Stat3 alone could transactivate HGF though Src augmented this effect. Increased phosphorylation of Stat3 tyrosine 705 was also observed in this line. Analysis of three ovarian cell lines revealed that Src/Stat3 expression was not able to activate the HGF promoter in two of these lines (SKOV3 and IOSE-80PC). Src/Stat3 expression did activate HGF transcription in OVCAR3 cells, but this effect was not mediated by the Stat3 site at nt-95. Stat3 phosphorylation at tyrosine 705 was observed in IOSE-80PC cells, but was insufficient to allow for activation of the HGF promoter. Human kidney (HEK293) and cervical carcinoma (HeLa) cells were also not Src/Stat3 permissive, despite high levels of Stat3 phospho-Y705. These results suggest that human breast cells are a uniquely permissive environment for HGF transactivation by Src/Stat3 which may allow for the inappropriate activation of HGF transcription during the early stages of breast transformation. This could lead to paracrine or autocrine activation of the Met receptor in breast carcinoma cells.

## Background

Hepatocyte Growth Factor (HGF) is a multi-functional cytokine primarily expressed in mesenchymal cells. By binding to its receptor Met, which is expressed on the surface of epithelial cells, HGF regulates many cellular processes including growth, angiogenesis, migration, morphogenesis, epithelial-mesenchymal transition and cell survival [1,2]. HGF participates in normal mammary development through tightly regulated paracrine signaling, however aberrant regulation of the HGF/Met signaling pathway is thought to contribute to tumorigenesis [3,4]. In addition, we [5] and others [6-8] have previously observed frequent over-expression of both HGF and Met in invasive human breast carcinomas.

The increased activation of Src and Signal Transducer and Activator of Transcription 3 (Stat3) proteins is associated with breast tumorigenesis [9,10] as well as a number of other human cancers [11,12]. Both activated Src and Stat3 mediate signals downstream of the HGF/Met pathway [13,14], and are key regulators of tumourigenic growth and survival [15,16]. In addition, Stat3 activation by Src induces specific gene regulation and is required for cell transformation [14,17]. We have previously shown that activated Src/Stat3 co-operativity enhances HGF transcriptional activity and protein expression, and induces HGF-dependent scattering in mammary epithelial cells [18,19]. We have also characterized a Src/Stat3 responsive region on the HGF promoter identifying a Stat3 consensus binding site at nt-95 that has been demonstrated to be required for HGF promoter activation by Src/Stat3 in mouse mammary carcinoma cells [18]. Overall, these observations suggest a model where positive feedback signals in the HGF/Met Src/Stat3 axis contribute to the transformation process.

The use of a murine system provides a useful model for the study of breast cancer, however the phenomenon observed in mouse mammary tumour cells has not been assessed in a human model, which may not necessarily recapitulate the disease in the same manner. Sequence homology comparison showed that the nt-95 Stat3 binding site as well as the sequences flanking this region is completely conserved between the mouse and human HGF genes. We therefore investigated the mechanisms of Src/Stat3 co-operativity and their interaction at the nt-95 site on the HGF promoter to activate HGF transcription in a panel of human epithelial cell lines. We demonstrated that human breast cells provide a permissive environment for HGF/Met signaling, and that the Stat3 nt-95 binding site is required to mediate the co-operative effects of Src/Stat3 induced HGF transactivation. These findings suggest a molecular mechanism that through co-operative SRC/Stat3 signaling could activate an HGF/Met autocrine loop in breast cancer cells.

## Results and discussion

**Figure 1:**
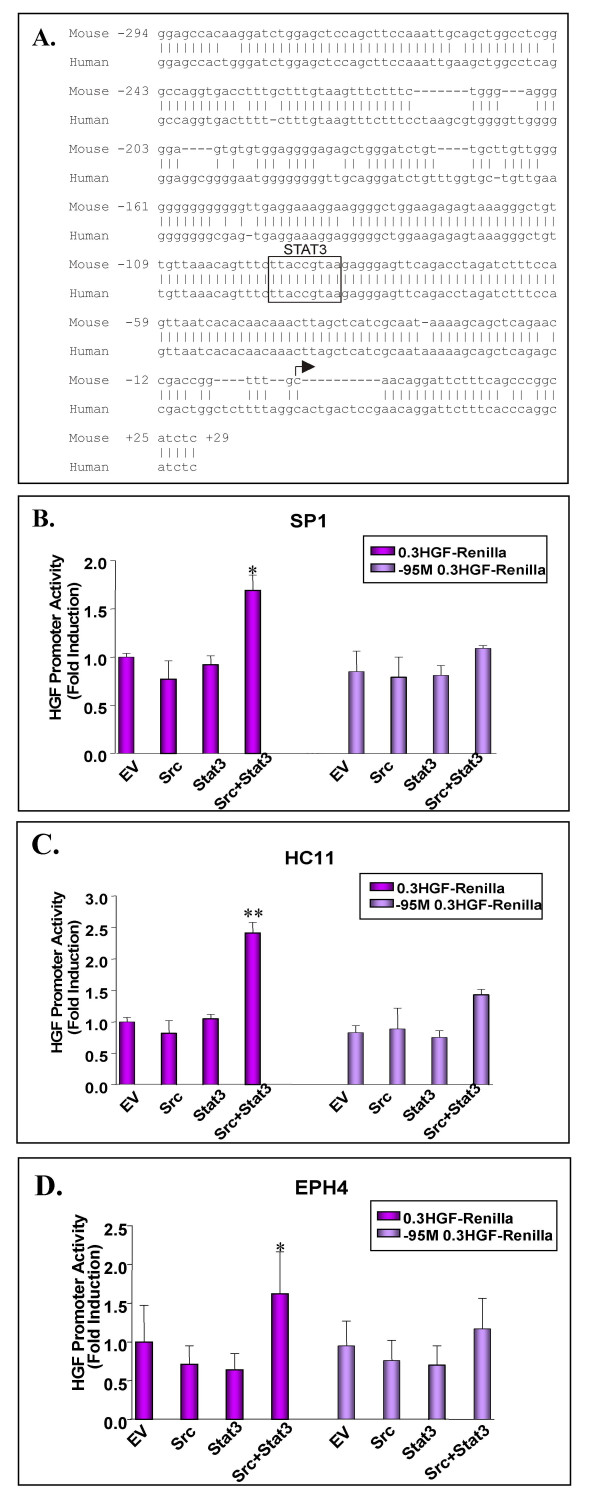
**Src/Stat3 mediated HGF promoter transactivation in mouse epithelial breast cell lines**. A DNA sequence alignment between the mouse HGF proximal promoter spanning from nt-274 to +29 is shown with the comparable region of the human gene (A). The Stat3 site at nt-95 is boxed and the transcriptional start site is indicated by an arrow. The 0.3 HGF-Renilla or -95 M 0.3 HGF-Renilla mouse proximal promoters were co-transfected with either activated Src or Stat3 alone, or in combination in: mouse mammary carcinoma SP1 cells (B), non-tumorigenic mouse mammary HC11 cells (C), and non-tumorigenic mouse mammary EPH4 cells (D). 48 hours post-transfection, cells were lysed and assayed for dual-luciferase activity. Values were normalized using a CMV-Luc internal control and are presented as fold induction relative to the empty vector (EV) for 0.3 HGF-Renilla. Asterisks indicate a significant increase in HGF promoter activity compared to the 0.3 HGF-Renilla empty vector control (*P *= 0.001*, *P *= 0.0005** using a Student's T-test). Transfections were repeated three times and values represent average results of triplicate samples +/- SD.

The ability of both Src and Stat3 to activate the HGF promoter was assessed by co-transfection of a mouse HGF proximal promoter construct into a variety of Src responsive mouse and human epithelial cell lines. The proximal promoter consisted of sequences from nt-274 to +29 of the mouse HGF gene cloned upstream of the renilla luciferase reporter gene, and shares approximately 83% homology to that of human HGF (Figure 1A). In addition to the wild-type reporter construct, we also generated a point mutant which eliminates the Stat3 recognition site located at nt-95. The Stat3 binding site, as well as the sequences flanking this region are completely conserved between the mouse and human genes.

**Figure 2:**
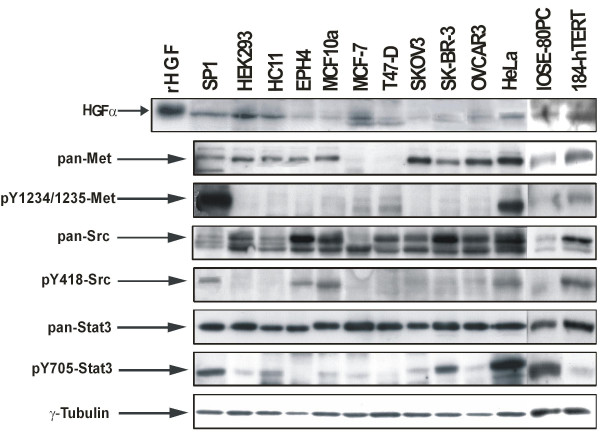
**Endogenous expression and activity of HGF/Met signaling proteins in mouse and human epithelial cell lines**. Endogenous protein expression and activation levels of HGF, Met, Src and Stat3 were characterized in mouse and human epithelial cell lines by western blot analysis. Whole cell lysates collected from the indicated cell lines were normalized and resolved on a reducing SDS-PAGE gel. Western blot analysis was performed using antibodies probing for the indicated proteins (arrows point to relevant band). γ-Tubulin was used as a loading control to normalize for overall protein concentration, and recombinant HGF (rHGF) served as a control for HGFα migration.

Co-transfection of activated Src or Stat3 alone had no effect on HGF promoter activity in mouse tumorigenic SP1 (Figure 1B), and non-tumorigenic HC11 and EPH4 (Figure 1C, D) breast cell lines. In contrast, co-transfection of both activated Src and Stat3 resulted in a 1.6 to 2.4-fold induction of HGF promoter activity depending on the cell line. This Src/Stat3 mediated HGF induction was severely diminished upon mutation of the nt-95 site on the HGF promoter, and indicates the necessity of this binding site for Stat3 mediated HGF activation. These observations extend our previous studies [18], investigating the activating functions of Src and Stat3 on HGF transcription in mouse breast cancer cells. Western blot analysis characterizing the patterns of endogenous expression of the proteins involved in the HGF/Met signaling pathway across a variety of epithelial cell lines indicate that the mouse mammary carcinoma SP1 cell line endogenously expresses high levels of HGF, while also expressing high levels of activated Met as indicated by Met receptor phosphorylation (Figure 2). Furthermore, the SP1 cell line also endogenously expresses high levels of activated Src and Stat3 as determined by Src and Stat3 phosphorylation, and is consistent with our previous findings which demonstrated the use of the SP1 cell line as an appropriate autocrine model for the study of co-operative Src/Stat3 signaling on HGF transcription in mice [19]. In contrast, EPH4 cells expressed very low levels of HGF, and both phosphorylated Met and Stat3. Detectable levels of HGF and phosphorylated Stat3 expression were observed in the HC11 cell line, however this was insufficient to cause phosphorylation of Met in these cells. This indicates that while tumourigenic SP1 cells may sustain a constitutively active HGF/Met signaling pathway, non-tumourigenic HC11 and EPH4 cells both represent a permissive environment for HGF transactivation upon appropriate Src/Stat3 activation.

**Figure 3:**
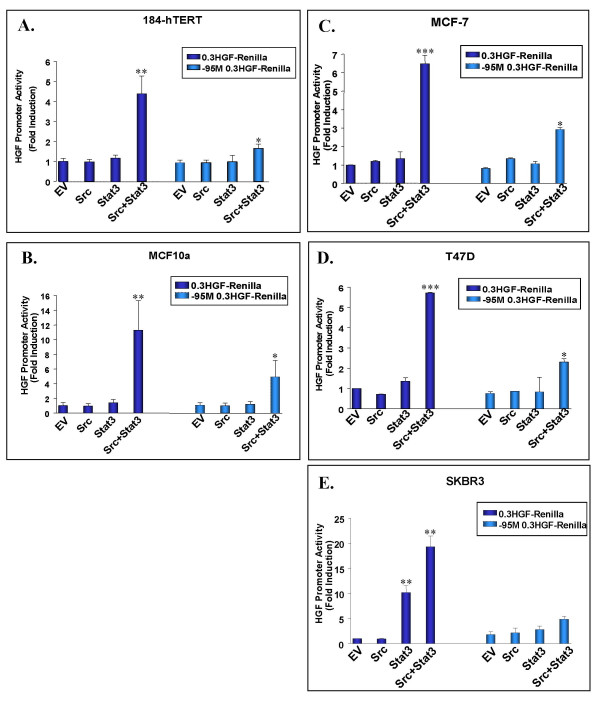
**Src/Stat3 mediated HGF promoter transactivation in human epithelial breast cell lines**. The 0.3 HGF-Renilla or -95 M 0.3 HGF-Renilla mouse proximal promoters were co-transfected with either activated Src or Stat3 alone, or in combination in: human non-tumorigenic 184-hTERT (A) and MCF10a breast cells (B), human MCF-7 adenocarcinoma breast cells (C), human T47-D ductal carcinoma breast cells (D), and human SK-BR-3 adenocarinoma breast cells (E). 48 hours post-transfection, cells were lysed and assayed for dual-luciferase activity. Values were normalized using a CMV-Luc internal control and are presented as fold induction relative to the empty vector (EV) for 0.3 HGF-Renilla. Asterisks indicate a significant increase in HGF promoter activity compared to the 0.3 HGF-Renilla empty vector control (*P *= 0.001*, 0.0003**, 0.0005*** using a Student's T-test). Transfections were repeated three times and values represent average results of triplicate samples +/- SD.

We next assessed human breast cell lines for HGF responsiveness in the presence of activated Src and Stat3. Strong Src and Stat3-dependent HGF promoter transactivation was observed in non-tumorigenic 184-hTERT and MCF10a breast cell lines (Figure 3A, B), as well as in the malignant MCF-7 adenocarcinoma and T47-D ductal carcinoma breast cell lines (Figure 3C, D), ranging from a 4 to 10-fold induction. In the highly malignant human SK-BR-3 adenocarcinoma breast cell line, transfection of Stat3 alone was sufficient to activate the HGF promoter by 10-fold, however upon co-transfection of both activated Src and Stat3, a 20-fold induction of HGF promoter transactivation was observed (Figure 3E). HGF induction was mediated through the nt-95 site on the HGF promoter since this activation was severely diminished upon its mutation in all the human breast cell lines surveyed.

Endogenous Src expression appears to be elevated in SK-BR-3 compared to MCF-7 and T47-D cells as indicated by western blot analysis. Although only moderate levels of Src Y418 phosphorylation were detected in SK-BR-3 cells (Figure 2), it is well established that these cells possess increased Src activity as a result of ErbB2 oncogene over-expression [20,21]. However, ErbB2 signaling in human breast cancer has been associated primarily with Src Y215 phosphorylation [22,23]. Therefore the apparent Src-independent activation of the HGF promoter in SK-BR-3 cells may be attributed to high levels of endogenously expressed Src pY215. SK-BR-3 cells also express high endogenous levels of activated Stat3 (Figure 2), though Stat3-dependent activation of the HGF promoter and an effect of both Src and Stat3 together is still observed. This finding indicates that the Src/Stat3 mediated activation of HGF requires levels of activated Stat3 expression above those of endogenously expressed Stat3. Interestingly, all of the human breast cell lines have similar high levels of Stat3, but HGF stimulation requires both activated Src and over-expression of Stat3. In fact, tissue microarray analysis of breast cancer tissues show elevated levels of both phosphorylated Y705-Stat3 and S727-Stat3 in 35% [24] and 62% [25] of cases respectively, indicating that the activation of Stat3 may play a key role in breast cancer oncogenesis. In addition, levels of both activated Stat3 and Src have been shown to be significantly higher in invasive breast carcinoma tissues suggesting the important role of elevated Src and Stat3 protein expression in malignant breast cancer progression [10].

Met receptor expression was widely observed in all cell lines with the exception of human MCF-7 and T47-D breast cell lines (Figure 2). It has previously been proposed that the co-operative effects of activated Src/Stat3 induced HGF expression result in the formation of an HGF autocrine loop, whereby epithelial cells secrete HGF and activate their own Met receptors maintaining a state of sustained HGF activation leading to tumorigenesis [5]. While the absence of Met receptor in these cells suggests that such an autocrine loop is not currently operating, this process may have played a role in the origin of these tumours. It is also possible that HGF-expressing carcinoma cells could "recruit" adjacent Met positive pre-malignant or malignant cells thereby stimulating these cells to invade.

**Figure 4:**
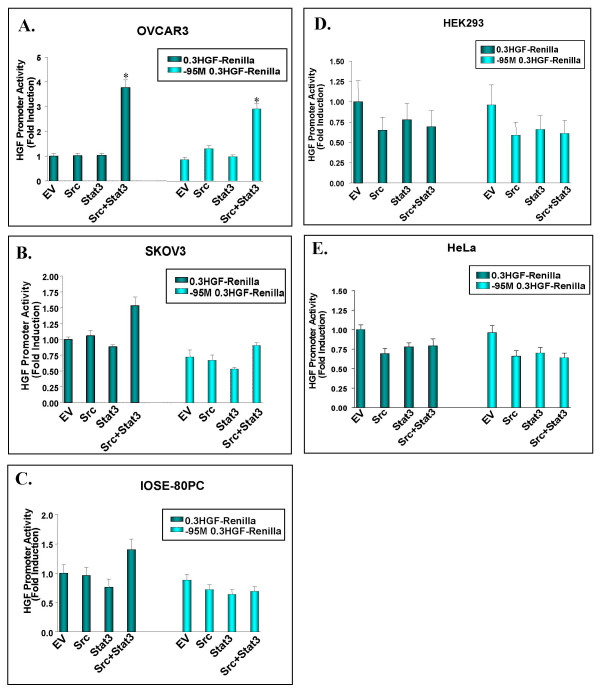
**Src/Stat3 mediated HGF promoter transactivation in human non-breast epithelial cell lines**. The 0.3 HGF-Renilla or -95 M 0.3 HGF-Renilla mouse proximal promoters were co-transfected with either activated Src or Stat3 alone, or in combination in: human ovarian tumorigenic OVCAR3 (A) and SKOV3 cells (B), human ovarian non-tumorigenic ovarian IOSE-80PC cells (C), human embryonic kidney HEK293 adenocarcinoma cells (D), and human cervical carcinoma HeLa cells (E). 48 hours post-transfection, cells were lysed and assayed for dual-luciferase activity. Values were normalized using a CMV-Luc internal control and are presented as fold induction relative to the empty vector (EV) for 0.3 HGF-Renilla. Asterisks indicate a significant increase in HGF promoter activity compared to the 0.3 HGF-Renilla empty vector control (*P *= 0.0005* using a Student's T-test). Transfections were repeated three times and values represent average results of triplicate samples +/- SD.

A number of other well established non-breast human lines such as ovarian, cervical and embryonic kidney epithelial cell lines were also surveyed to assess HGF responsiveness in the presence of activated Src and Stat3. Co-transfection of activated Src and Stat3 induced a 4-fold activation of the HGF promoter in the tumorigenic ovarian OVCAR3 cell line, however this effect does not appear to occur via the nt-95 site since mutation of this site showed no inhibitory effect on HGF induction (Figure 4A). Western blot analysis indicates that the OVCAR3 cell line expresses relatively low levels of endogenous activated Src and Stat3, and they do not appear to over-express any of the proteins involved in the HGF/Met signaling pathway (Figure 2). The observed activation of both the wild-type and mutated HGF promoters may reflect the activation of other transcription factors by Src/Stat3 which in turn activate the HGF promoter through an alternate site.

It is interesting that this alternate pathway of HGF transcriptional activation was only observed in one ovarian cell line suggesting that a unique HGF related pathway may exist in these cells. The OVCAR3 cell line was the only line to exhibit this characteristic as ovarian tumorigenic SKOV3, non-tumorigenic IOSE-80PC and HEK293 kidney adenocarcinoma cell lines did not support transactivation of the HGF promoter upon co-transfection of activated Src and Stat3 (Figure 4B–D). Similarly, transactivation of the HGF promoter was not observed upon co-transfection of activated Src and Stat3 in HeLa cervical carcinoma cells (Figure 4E) despite the high levels of activated Stat3 and Met expressed in this cell line (Figure 2). This finding parallels the expression patterns observed in SP1 cells which are Src/Stat3 responsive indicating that over-expression of these proteins is not sufficient for HGF transactivation in HeLa cells. This suggests that breast cells must possess a mechanism which produces a permissive environment for Src/Stat3 mediated HGF transcriptional activation.

## Conclusion

We observed activated Src/Stat3 mediated HGF transactivation in both malignant and non-tumorigenic human breast cell lines but not in human non-breast epithelial cell lines. This suggests that the activated Src/Stat3 mediated effects are tissue-specific with breast cell lines providing a permissive environment for the HGF/Met signaling pathway. Furthermore, in addition to requiring the over-expression of both activated Src and Stat3 to induce HGF transcription, the Stat3 nt-95 binding site on the HGF promoter is also a necessary requirement to mediate the co-operative Src/Stat3 transactivation of the HGF promoter in human breast cells. These studies suggest that the HGF/Met Src/Stat3 signaling loop may be a potential treatment target and/or a prognostic indicator for invasive breast cancer.

## Methods

### Mutagenesis and cloning

0.3 HGF-Renilla and -95 M 0.3 HGF-Renilla constructs were generated by PCR using 0.5 HGF-Luc and -95 M 0.5 HGF-Luc constructs [18] respectively as HGF template DNA. The HGF 5' Forward (*5'-GGGCTCGAGGGAGCCACAAGGATC-3'*) and HGF 3' Reverse (*5'-GGGAAGCTTGAGATGCCGGGCTG-3'*) primers were used to generate the 0.3 HGF fragment were inserted into the pRL-null vector using *Xho*I and *Hind*III restriction sites.

### Transient transfection and dual luciferase assay

Mouse and human cells were cultured as described in Additional file 1. SP1 and HC11 cell line transfections were performed using the LipofectAMINE Plus™ system (Invitrogen™) according to manufacturer's protocols. Cells were seeded in 12-well plates at cell densities indicated [See Additional file 1], incubated overnight in complete growth medium, and transfected with a total of 1 μg of DNA. MCF10a cell line transfections were performed using ESCORT V Transfection Reagent (Sigma Aldrich, Oakville, ON, Canada) according to manufacturer's protocols. Cells were seeded in 12-well plates at cell densities indicated [See Additional file 1], incubated overnight in complete growth medium, and transfected with a total of 1.4 μg of DNA. All other cell line transfections were performed using FuGene 6 Transfection Reagent (Roche Applied Sciences, Laval, QC, Canada) according to manufacturer's protocols. Cell lines were seeded in 12-well plates at cell densities indicated [See Additional file 1], incubated overnight in complete growth medium, and transfected with a total of 250 ng of DNA.

For luciferase assays, cells were lysed in 1× Passive Lysis Buffer (Promega) 48 hours post-transfection, and Dual-Luciferase™ Reporter assays (Promega) were performed according to manufacturer's protocols using an EG&G Berthold microplate luminometer. All luciferase transfections were normalized to a corresponding CMV-luciferase internal control (Promega) to adjust for variations in transfection efficiency between different cell lines.

### Western Blotting

Cells were washed twice with 1× PBS and lysed in RIPA buffer (50 mM Tris-HCl pH 7.4, 150 mM NaCl, 1 mM EDTA, 1% NP-40, 0.25% Sodium deoxycholate, 1 μg/mL Trasylol, 1 μg/mL Leupeptin, 1 mM DTT, 1 mM phenylmethylsulfonyl fluoride, 1 mM NaF, 1 mM Na_3_VO_4_, and 20 mM β-glycerophosphate). Collected lysates were incubated for 15 minutes at 4°C and subjected to protein determination using a Bio-Rad Dc protein assay kit (BioRad, Mississauga, ON, Canada). Normalized protein levels were resolved on an 8 or 10% reducing SDS-PAGE gel (with 3% β-2-mercaptoethanol in loading buffer), and transferred onto PVDF membranes (Millipore). Separate membranes were probed with the indicated primary antibodies, anti-Stat3 (#9132), phospho-specific anti-Stat3(pY705) (#9131), and anti-Met(pY1234/1235) (#3126) (Cell Signaling Technology, Beverly, MA, USA), anti-human Met (C12), anti-mouse Met (B2), anti-Src (B12), anti-HGFα (H-145) (Santa Cruz Biotechnology Inc, Santa Cruz, CA, USA), phospho-specific anti-Src(pY418) (#44-660A1) (Biosource International, Burlington, ON, Canada), and anti-γ-tubulin (GTU-88) (Sigma Aldrich, Oakville, ON, Canada). Immune complexes were detected using horse-radish peroxidase (HRP)-labelled anti-rabbit or anti-mouse IgG (Amersham Biosciences, Baie d'Urfè, QC, Canada) followed by enhanced chemi-luminescence (Perkin Elmer, Woodbridge, ON, Canada).

## Competing interests

The author(s) declare that they have no competing interests.

## Authors' contributions

BEE and CRM conceived the study, MS carried out all of the experiments and all three authors contributed to the writing of the article.

## Supplementary Material

Additional file 1 Human and Mouse Cell Lines and Culture Conditions. The various cell lines, with their culture and transfection conditions are described. Click here for file
