# Supplementary material for: A novel activating role of SRC and STAT3 on HGF transcription in human breast cancer cells
Source: Mol Cancer. 2007 Oct 29;6:69. doi: 10.1186/1476-4598-6-69 (PMC2173908; doi:10.1186/1476-4598-6-69)
Supplement: Additional file 1 — Human and Mouse Cell Lines and Culture Conditions. The various cell lines, with their culture and transfection conditions are described. [file 1476-4598-6-69-S1.doc]

**Table S1: Human and Mouse Cell Lines and Culture Conditions**

| **Cell Line** | **Source** | **Origin** | **Culture Conditions** | **Transfection**  **Cell Density (per mL)** |
| --- | --- | --- | --- | --- |
| HC11 | Gift from  D. Medina | Mouse (Balb/c-COMMA-D)  Non-tumorigenic Mammary Epithelial [1] | RPMI-1640,  10% FBS  1X Pen/Strep  5ug/mL Insulin  10ng/mL EGF | 1x10^5^ |
| EPH4 | Gift from  C. Roskelly | Mouse  Non-tumorigenic Mammary  Epithelial [2] | DMEM/F12,  5% FBS  1X Pen/Strep  5ug/mL Insulin | 1x10^5^ |
| SP1 | B. Elliott | Mouse Mammary  Epithelial Carcinoma [3] | RPMI-1640,  10% FBS  1X Pen/Strep | 1x10^5^ |
| 184-hTERT | Gift from  C. Roskelly | Human  Non-tumorigenic Mammary  Epithelial [4] | MEBM  1X Pen/Strep  400ug/mL G418/neo  1ug/mL Transferrin  5ng/mL Isoproterenol  Single Quot | 5x10^4^ |
| MCF10a | ATCC  Manassas, VA | Human  Non-tumorigenic Mammary  Epithelial [5] | DMEM/F12, 5% HS  1X Pen/Strep  10ug/mL Insulin  100ng/mL CT  20ng/mL EGF  0.5ug/mL HC | 5x10^4^ |
| MCF-7 | ATCC  Manassas, VA | Human Mammary  Epithelial Adenocarcinoma | RPMI-1640,  10% FBS  1X Pen/Strep | 1x10^5^ |
| T47-D | ATCC, Manassas, VA | Human Mammary  Epithelial Ductal Carcinoma | RPMI-1640,  10% FBS  1X Pen/Strep | 1x10^5^ |
| SK-BR-3 | ATCC, Manassas, VA | Human Mammary  Epithelial Adenocarcinoma | DMEM, 10% FBS  1X Pen/Strep | 1x10^5^ |
| IOSE-80PC | Gift from  C. Roskelly | Human Non-tumorigenic  Ovarian Epithelial [6] | DMEM, 5% FBS  1X Pen/Strep | 1x10^5^ |
| OVCAR3 | ATCC, Manassas, VA | Human  Tumorigenic Ovarian  Epithelial | RPMI-1640,  10% FBS  1X Pen/Strep  10ug/mL Insulin | 1x10^5^ |
| SKOV3 | ATCC, Manassas, VA | Human Tumorigenic Ovarian Epithelial | DMEM, 5% FBS  1X Pen/Strep | 1x10^5^ |
| HEK293 | ATCC, Manassas, VA | Human Kidney  Epithelial Adenocarcinoma | DMEM, 10% FBS  1X Pen/Strep | 1x10^5^ |
| HeLa | ATCC, Manassas, VA | Human Cervical  Epithelial Carcinoma | DMEM, 10% FBS  1X Pen/Strep | 5x10^4^ |

References

1. Ball R, Friis R, Schroenenberger C, Doppler W, Groner B: **Prolactin regulation of b-casein gene expression and of cytosolic 120-kd protein in a cloned mouse mammary epithelial cell line.** *EMBO Journal* 1988, **7:**2089-2095.

2. Barahona M, Fialka I, Gonzalez-Sancho J, Asuncion M, Gonzalez M, Iglesias T, Bernal J, Beug H, Munoz A: **Thyroid hormone regulates stromelysin expression, protease secretion and the morphogenetic potential of normal polarized mammry epithelial cells.** *EMBO Journal* 1995, **14:**1145-1155.

3. Elliott B, Tam S, Dexter D, Chen Z: **Capacity of adipose tissue to promote growth and metastasis of a murine mammary carcinoma: effect of estrogen and progesterone.** *International Journal of Cancer* 1992, **51:**416-424.

4. Nijjar T, Wigington C, Garbe J, Waha A, Stampfer M, Yaswen P: **p57KIP2 expression and loss of heterozygosity during immortal conversion of cultured human mammary epithelial cells.** *Cancer Res* 1999, **59:**5112-5118.

5. Soule H, Maloney T, Wolman S, Peterson W, Brenz R, McGrath C, Russo J, Pauley R, Jones R, Brooks S: **Isolation and characterization of a spontaneously immortalized human breast epithelial cell line, MCF-10.** *Cancer Res* 1990, **50:**6075-6086.

6. Choi JH, Park SH, Leung PCK, Choi KC: **Expression of Leptin Receptors and Potential Effects of Leptin on the Cell Growth and Activation of Mitogen-Activated Protein Kinases in Ovarian Cancer Cells.** *J Clin Endocrinol Metab* 2005, **90:**207-210.
